# Effects of lower extremity neuromuscular and mechanical characteristics on running economy and sports performance of long-distance runners under different relative paces

**DOI:** 10.3389/fbioe.2026.1752244

**Published:** 2026-02-12

**Authors:** Zhengze Tan, Zihao Li, Yiling Ding, Dantong Wang, Yongan Wang

**Affiliations:** 1 Capital University of Physical Education And Sports, Institute of Physical Education and Training, Beijing, China; 2 College of Physical Education, China West Normal University, Nanchong, China; 3 College of Physical Education, Hebei Normal University, Shijiazhuang, China

**Keywords:** middle and long-distance running, running economy, sports performance, strength quality, surface electromyography

## Abstract

**Objective:**

To explore the potential correlational patterns between lower extremity explosive power, strength balance, joint stiffness, neuromuscular characteristics and running economy (RE) as well as personal best (PB) of long-distance runners under different relative paces, so as to provide a preliminary theoretical clues for optimizing endurance training.

**Methods:**

Ten male second-class long-distance runners were recruited. Under the paces of PB70%, PB80% and PB90%, RE, maximal oxygen uptake (VO_2_max), lower extremity explosive power (CMJ, SJ, EUR, RSI), joint peak torque ratio (PTR), joint stiffness and neuromuscular indicators (RMS, CAR) were tested using gas metabolism analysis, force platform, isokinetic strength testing system, motion capture system and electromyography equipment. Data were processed by one-way repeated measures analysis of variance, paired samples t-test and Pearson correlation analysis.

**Results:**

RE increased significantly with the increase of pace (p < 0.05), and the correlation between RE and PB at PB80% and PB90% was stronger than that between VO_2_max and PB; RSI was significantly negatively correlated with RE70%, RE90% and PB (p < 0.05), while CMJ, SJ and EUR had no significant correlation; at 180°/s angular velocity, the hip joint flexor eccentric-extensor concentric PTR was significantly positively correlated with RE70%, RE90% and PB, and the knee joint flexor concentric-extensor eccentric PTR was significantly positively correlated with RE at all paces and PB (p < 0.05); knee joint stiffness was significantly negatively correlated with RE at all paces (p < 0.05); the RMS of vastus medialis (VM) was significantly positively correlated with RE70% and RE90%, and the knee joint CAR was significantly negatively correlated with RE at all paces (p < 0.05).

**Conclusion:**

This study is strictly exploratory and hypothesis-generating. RE is a core indicator potentially associated with long-distance running performance. RSI, specific hip and knee PTR at 180°/s angular velocity, knee joint stiffness and knee joint neuromuscular activation characteristics (VM RMS, knee joint CAR) show potential correlational associations with RE. The associations between these indicators and RE are pace-dependent and joint-specific, which can provide preliminary scientific reference for generating hypotheses about long-distance running training.

## Introduction

Running Economy (RE) is one of the key indicators to measure endurance sports performance, defined as the energy consumption or oxygen uptake required to maintain a certain running speed under submaximal intensity (Bassett and Howley, 2000). RE represents the translation of energy turnover into running velocity and is cited as a stronger indicator of endurance performance than VO_2_max alone within athletically homogenous populations (Conley and Krahenbuhl, 1980). Improving RE not only means consuming less energy at the same exercise intensity, but also reflects the optimization of individuals in neuromuscular coordination and energy transfer efficiency (Morgan et al., 1989). Revealing the neuromuscular and mechanical mechanisms related to RE is an important scientific issue for understanding running performance. Existing studies have shown that RE is jointly affected by multiple factors such as muscle explosive power, strength balance, joint stiffness, muscle activation patterns and neural control strategies (Li et al., 2019). However, there are still many inconsistent views in the literature on the relationship between these indicators and RE.

Lower extremity explosive power is an important indicator reflecting the stretch-shortening cycle (SSC) ability and energy conversion efficiency of the tendon-muscle system (Radnor et al., 2018). During the rapid eccentric-concentric transition process, muscles and tendons store and release energy in the form of elastic potential energy, which is also crucial in long-distance running. Countermovement Jump (CMJ) and Squat Jump (SJ) are commonly used to evaluate the efficiency of SSC. The elastic utilization ratio (EUR) corresponding to their difference and the reactive strength index (RSI) can quantitatively reflect the energy storage and feedback characteristics of the muscle-tendon system (Flanagan and Comyns, 2008; Bosco et al., 1981). Existing studies have shown that higher EUR or RSI is usually associated with lower metabolic cost and better RE, but the relevant conclusions are inconsistent (Radnor et al., 2018). Some scholars have found that the CMJ of high-level runners is indeed better than that of ordinary trainers (Rebelo et al., 2022), while others have pointed out that the relationship between CMJ and RE varies at different paces, and there may be no significant correlation under high-speed conditions (Spurrs et al., 2003). This contradiction may be due to differences in individual tendon compliance, running posture and speed regulation strategies. Some studies believe that under high-speed conditions, the utilization of elastic energy depends more on the timing of neural control than on the mechanical properties of tendons alone (Ar et al., 2006). The combined analysis of EUR and RSI is helpful to comprehensively evaluate the characteristics of elastic energy utilization in running and its real contribution to RE.

Corresponding to the explosive power characteristics is the strength balance relationship between different muscle groups of the lower extremities, which is often reflected by joint peak torque (PT) and PTR. PTR usually refers to the moment ratio of flexor and extensor muscles at different angular velocities, and is a key indicator to measure joint control stability and strength symmetry (Dauty et al., 2003). Studies have shown that a reasonable PTR can reduce exercise energy waste and injury risk, while imbalance may lead to joint stress concentration and decreased exercise efficiency (Whitney et al., 2025). Some studies have found that high-level runners have more stable torque output at different angular velocities (such as 60°/s, 180°/s and 300°/s), indicating that they can maintain a relatively ideal strength ratio and coordination, thereby optimizing RE (Sundby and Gorelick, 2014). However, other results show that the correlation between PTR and RE is not fixed, and factors such as training methods, muscle group endurance and neural feedback regulation may cause differences (Guney et al., 2016). In addition, some studies believe that PTR at medium angular velocity may better represent the dynamic stability level in long-distance running, while the decrease in strength output or imbalance in proportion under high-speed conditions will lead to reduced energy conduction efficiency (Duhig et al., 2019). Therefore, analyzing the changes of PT and PTR at multiple angular velocities is helpful to reveal the coupling mechanism between strength balance and RE.

There are significant differences in the requirements for the coordinated work of lower extremity flexor and extensor muscles between the stance phase (accounting for approximately 60% of the gait cycle) and swing phase (about 40%) of running gait (Sever et al., 2024). During the early stance phase (from landing to mid-stance), the hip and knee flexors undergo eccentric contraction to buffer ground impact forces, while the extensors are pre-activated to store elastic potential energy; in the late stance phase (from mid-stance to toe-off), propulsive force is provided by the concentric contraction of extensors, and the swing phase requires the coordination of concentric contraction of flexors and eccentric contraction of extensors to complete the forward swing and deceleration of the lower extremities (Leporace et al., 2023). The 180°/s angular velocity is close to the average hip/knee flexion-extension velocity during running, matching the muscle’s SSC working mode critical for elastic energy utilization. The joint flexor eccentric-extensor concentric PTR at 180°/s directly corresponds to the muscle torque coordination efficiency during the “buffer-propulsion” transition in the stance phase—a reasonable ratio can reduce energy loss during the buffer phase and improve energy conduction efficiency during the propulsion phase; the knee joint flexor concentric-extensor eccentric PTR is related to the connection between “forward swing-deceleration” in the swing phase and propulsion in the late stance phase, and its optimization can reduce redundant joint movement and metabolic cost (Inostroza-Ríos et al., 2025).

At the joint mechanical level, joint stiffness is an important parameter describing the absorption and feedback of ground impact energy by the lower extremities during running. Moderate stiffness helps to store elastic potential energy and improve mechanical efficiency, but excessive stiffness leads to insufficient impact absorption and increased muscle metabolic burden (Liu et al., 2022). Some studies have found that runners increase lower extremity stiffness to reduce energy loss when the pace increases (Dumke et al., 2010), while others point out that a more flexible gait can improve stride economy, thereby improving RE (Struzik et al., 2021). This difference indicates that the relationship between stiffness and RE is not monotonically changing, and there may be an optimal distribution dependent on speed. Due to differences in stiffness calculation methods (angle-moment or displacement-reaction force model) and test populations in different studies, the specific mechanism of joint stiffness on running energy efficiency needs further exploration.

At the same time, the electrophysiological characteristics of muscles reflect the driving strategy and energy regulation level of the nervous system. The root mean square (RMS) of electromyography (EMG) signals is often used to reflect muscle fiber recruitment and activation intensity (Türker and Sze, 2013). Usually, as the speed increases, RMS gradually increases, reflecting higher muscle output power. But excessively high RMS also means increased metabolic energy consumption (Hortobágyi et al., 2011). Studies have found that experienced runners can achieve the same or higher output with lower RMS at different speeds, which reflects better neuromuscular coordination efficiency (Tam et al., 2019). However, the relationship between RMS and RE is inconsistent among different muscle groups and individuals, suggesting that there may be individual differences in neural driving strategies.

The muscle co-activation ratio (CAR) further reveals the coordination characteristics between agonist and antagonist muscles. A low CAR is often regarded as a manifestation of the nervous system inhibiting the ineffective activities of antagonist muscles, thereby reducing energy waste and improving RE (Saunders et al., 2004). But some scholars point out that moderate co-activation is helpful to improve joint stability and coordination during high-speed or long-duration running (Jiang et al., 2016). Therefore, the “optimal level” of CAR may change with pace and muscle load. Excessively low co-activation may cause unstable gait or disordered movement rhythm, while excessively high co-activation increases metabolic consumption. Different studies have drawn opposite conclusions due to differences in muscle group selection and algorithms, indicating that its energy efficiency effect is still controversial.

Overall, existing studies lack consistent understanding of the contributions of lower extremity explosive power, strength balance, joint stiffness and neuromuscular characteristics to RE. Based on this, this exploratory study took long-distance runners as the research objects, measured lower extremity explosive power characteristics (CMJ, SJ, RSI, EUR), joint strength balance characteristics (PT, PTR), joint stiffness, muscle activation intensity (RMS) and co-activation ratio (CAR) under different relative paces (PB 70%, 80%, 90%), and conducted a comprehensive analysis combined with RE and personal best (PB). The purpose of this study is to generate hypotheses about the balance mechanism of how the neuromuscular system regulates strength output, joint stability and energy efficiency under different speeds, and to provide a preliminary theoretical basis for endurance training and running technology optimization.

## Methods

### Participants

The sample size was determined beforehand using G*Power (version 3.1.9.7) software. Based on a repeated-measures analysis of variance ANOVA with a large effect size (f = 0.8), α = 0.05, and power = 0.8, a minimum of 10 participants was required (Li et al., 2025). Ten male second-class athletes specializing in 5000 m or 10000 m were recruited from Capital University of Physical Education and Sports. The participants’ age was 20.2 ± 2.1 years, height was 175.9 ± 2.6 cm, and weight was 64.5 ± 3.5 kg. The participants’ weekly running volume in the past 3 months was more than 30km, and they had no lower extremity injuries. To avoid fatigue, the participants did not perform strenuous exercise within 24 h before the experiment. All participants signed a written informed consent form before the experiment. This study has been approved by the Ethics Committee of Capital University of Physical Education and Sports (approval number: 2024A121).

### Protocol

Participants were required to undergo a 2-day test, with an interval of no less than 72 h between the two tests. The first day’s test included RE and VO_2_max tests. The second day’s test included maximal voluntary contraction (MVC) test, lower extremity explosive power test, lower extremity joint strength test, and lower extremity stiffness and EMG test. To eliminate the confounding effect of running shoes on the test results, participants were required to wear a standardized pair of neutral cushioned running shoes (Nike Air Zoom Pegasus 34) provided by the laboratory.

### Aerobic capacity test

The participants’ 10000 m best performance in training and competitions in the past year was regarded as their personal best (PB), and the percentage of the best performance pace was used as the speed for RE evaluation (PB was 1991.75 ± 47.43s). First, the participants performed a warm-up at a self-selected speed on a treadmill (Precor TRM833, USA) for no less than 10 min and stretching for 5 min. After completion of the warm-up, the participants performed a three-stage interval running protocol, during which respiratory and metabolic parameters were recorded using a portable gas metabolic analysis system (META-LYZER 3B-R2, Cortex, Germany), while heart rate metrics were monitored with a Polar heart rate belt. The speeds were 70%, 80% and 90% of the best performance, each stage lasted 4 min with an interval of 5 min (Thibault et al., 2025). RE was defined as the average oxygen uptake (VO_2_) in the last 1 min of each stage. After the RE test, the VO_2_max test was performed. The treadmill speed was set to 17 km/h (about 95% of the best performance), and the speed increased by 1 km/h every 2 min until the participants felt exhausted and stopped the test. The prerequisite conditions for conducting a VO_2_max test are as follows: (1) A rest period of no less than 20 min; (2) A heart rate that does not exceed 10% of the pre-test value; (3)Confirmation from the participants that they are in a good state and ready to proceed with the test. The VO_2_max judgment criteria were: (1) RPE was 19 or 20; (2) HRmax≥(220-age)+15; respiratory quotient (RQ)≥1.05; (3) VO_2_ plateaued and no longer increased with the increase of exercise intensity.

Prior to each test, the portable gas metabolism system (META-LYZER 3B-R2, Cortex, Germany) was calibrated in accordance with the manufacturer’s instructions, including two-point gas calibration with a certified reference gas mixture and room air, volume/flow calibration with a 3-L syringe, and input of ambient temperature, barometric pressure and humidity. Calibration was repeated if deviations exceeded the manufacturer’s acceptable limits.

### MVC test

**FIGURE 1:**
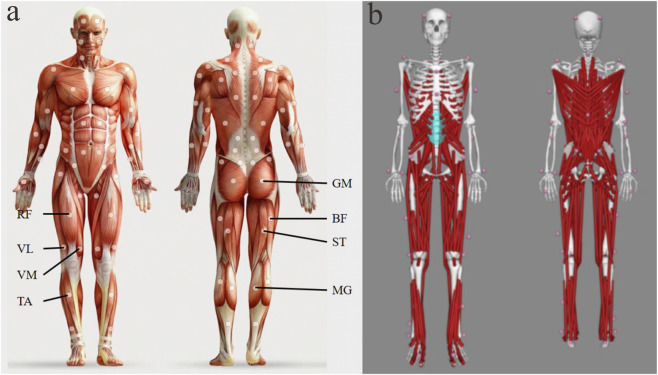
Schematic illustration of electrode locations and marker placement. **(a)**Electrode placement; RF = Rectus femoris; VL = Vastus lateralis; VM = Vastus medialis; TA = Tibialis anterior; GM = Gluteus maximus; BF = Biceps femoris; ST = Semitendinosus; MG = Medial gastrocnemius; **(b)** Marker locations; Pink circles indicate the positions of the markers.

Prior to the formal EMG recordings, maximal voluntary contractions (MVCs) of the target muscles were assessed for subsequent EMG normalization. EMG (Delsys Trigno, United States) was used to record muscle activity, and eight sensor units were placed over the rectus femoris (RF), vastus lateralis (VL), vastus medialis (VM), tibialis anterior (TA), gluteus maximus (GM), biceps femoris (BF), semitendinosus (ST), and medial gastrocnemius (MG) of the dominant lower limb (Figure 1a). For each muscle, participants performed 3 MVC trials (each lasting 3–5 s) with a 60-s rest interval between trials to avoid fatigue. The MVC protocol was standardized as follows: (1) RF, VL, and VM: seated knee extension against manual resistance; (2) TA: seated ankle dorsiflexion against manual resistance; (3) GM: prone hip extension against manual resistance; (4) BF and ST: seated knee flexion against manual resistance; and (5) MG: standing ankle plantarflexion against manual resistance.

For data analysis, EMG signals of each muscle were normalized to the highest EMG amplitude obtained across three MVC trials for that muscle.

### Lower extremity explosive power test

It included CMJ and SJ tests (Barnes et al., 2014). In the CMJ test, the participants were required to stand on a force platform (Kistler 9281CA, Switzerland, frequency 1000 Hz) with hands on hips, and quickly complete knee flexion squat-vertical jump-fall to the force platform after being ready. In the SJ test, the participants squatted with hands on hips, knees flexed to 90° and half-squatted on the force platform, and quickly jumped upward and fell to the force platform after being ready (no pre-swing action was allowed before jumping). For both tests, participants were required to perform two practice jumps and three formal tests (interval no less than 1 min).

### Lower extremity joint peak torque test

A multi-functional isokinetic strength testing and training system (Isomed 2000; Germany) was used to test the peak torque of the flexor and extensor muscles of the hip, knee and ankle joints. The test speeds were 60°/s (representing maximal strength capacity) and 180°/s (representing speed strength capacity), each speed was repeated 5 times with an interval of 30 s (Hayes et al., 2004). The test was performed on the dominant hip, knee and ankle joints. After the 60°/s speed test, the 180°/s test was performed with an interval of 1 min.

In this study, only the dominant limb (defined as the leading limb for push-off during running) was tested, mainly based on the following considerations: First, long-distance running is a symmetric periodic movement, and the movement pattern and torque output characteristics of the dominant limb can representatively reflect the overall motor strategy (Barnes et al., 2014). Existing studies have confirmed that in athlete populations with homogeneous training backgrounds, the difference in PTR between the dominant and non-dominant limbs is not statistically significant (Ren et al., 2025); second, focusing on testing the dominant limb can reduce test duration and subject fatigue, avoiding the interference of multi-limb testing on the accuracy of subsequent data.

### Lower extremity stiffness and EMG test

After the participants performed a warm-up on the treadmill for no less than 10 min and stretching for 5 min, they practiced on the treadmill at 70%, 80% and 90% of the best performance speed (PB70%, PB80%, PB90%) respectively. After the participants felt familiar with various speeds, the formal test was performed on the track. Each speed was practiced 2 times and formally tested 3 times. A successful test required the subject’s dominant foot to fully step on the force platform, and the data closest to the target speed was taken for subsequent analysis. During the test, a motion capture system (OptiTrack, 8-camera, NaturalPoint, USA) was used to collect the subject’s kinematic information, using the system’s Conventional Full Body 39-marker model for marker placement (Figure 1B); a force platform system was used to record the ground reaction force (GRF) of the subject’s dominant side; EMG was used to collect the subject’s muscle electrical activity, and synchronized collection was performed with the motion capture system and force platform. A portable speed measurement system (Smart Speed; Fusion Sport, Brisbane, Australia) was used to monitor the average running speed 3 m before and after the force platform, and the acceptable deviation was 5%.

### Data preprocessing

#### GRF data

The 3-axis GRF of the force platform was subjected to 30 Hz low-pass filtering (fourth-order, Butterworth) for subsequent analysis. In the lower extremity explosive power test, the moment when the first vertical axis GRF (F_y_) <10N in the air after the start of the test and take-off was recorded as the take-off moment t_1_, and the moment when the first F_y_ >10N after t_1_ was recorded as the landing moment t_2_. In the CMJ test, the moment when F_y_ inflected during squatting to take-off after the start of the test was recorded as the squat lowest point moment t_0_. In the lower extremity stiffness and EMG test, the moment when the first F_y_ >10N was recorded as the ground contact moment t_1_, and the moment when the first F_y_ <10N was recorded as the take-off moment t_2_.

#### Motion capture data

This study used OpenSim 4.4 version for kinematic analysis. First, the original motion capture data (C3D format) was converted to OpenSim-compatible.mot format. The data was truncated according to t_1_ and t_2_ moments. Then, model scaling was performed based on the Full-body Musculoskeletal Model of the Lumbar Spine (FBLS), and the model was adjusted to match the anthropometric characteristics of each participant (height, weight, and segment length calculated according to the marker position). This process was completed using the “Scale Model” tool with the following thresholds: total marker position error <4 cm, root mean square (RMS) error <2 cm. Then, the “Inverse Kinematics” tool was used to calculate the time change information of joint angles, and the results were subjected to 6Hz low-pass filtering (fourth-order, Butterworth). Combined with the preprocessed external force data, inverse dynamics analysis was performed to obtain the time change information of joint moments.

#### EMG data

The data was truncated according to t1 and t2 moments. The truncated EMG data was filtered with a fourth-order Butterworth band-pass filter (cut-off frequency set at 10–400 Hz). Then, the linear envelope was obtained using a moving RMS window with a width of 0.125 s and no overlap. Finally, the EMG signals were normalized to the maximum value observed for each muscle across MVC tests.

#### Analysis indicators

In the aerobic capacity test, the analysis indicators included VO_2_max (mL/kg/min), RE at 70%, 80% and 90% of the best performance speed, which were RE70%, RE80% and RE90% respectively.

In the lower extremity explosive power test, the analysis indicators included CMJ (cm), SJ (cm), EUR (%) and RSI (m/s). For CMJ and SJ, the highest value of the formal test was selected for analysis, and the calculation formula was:

$$h=\frac{1}{8}g\times {\left(t_{2}‐t_{1}\right)}^{2}$$ (1)

In Formula 1, g is the gravitational acceleration, taken as 9.8 m/s^2^, t2 is the landing moment, and t1 is the take-off moment.

For EUR, the calculation formula was:

$$\text{EUR}=\frac{CMJ‐SJ}{CMJ}\times 100\%$$ (2)

In Formula 2, CMJ and SJ represent the CMJ height and SJ height respectively.

For RSI, this study adopted the CMJ-derived RSI, with the calculation formula as follows:

$$\text{RSI}=\frac{CMJ}{t_{1}‐t_{0}}\times 100\%$$ (3)

In Formula 3, CMJ represents the CMJ height, t_1_ represents the take-off moment, and t_0_ represents the squat lowest point moment.

In the lower extremity joint strength test, the analysis indicators included the PTR representing static joint strength balance (flexor concentric-extensor concentric; flexor eccentric-extensor eccentric) and the PTR representing dynamic joint movement (flexor eccentric-extensor concentric; flexor concentric-extensor eccentric).

In the lower extremity stiffness and EMG test, the analysis indicators included the average joint stiffness (N·m/kg/°) of the hip, knee and ankle joints, muscle RMS and joint muscle co-activation ratio (CAR).

For the purpose of this study, stance-phase joint stiffness was focused on, as it directly relates to the key functional phases of impact buffering and propulsive force generation. This phase was defined from initial ground contact (t_1_: first vertical GRF Fy >10N) to toe-off (t_2_: first Fy <10N). Whole-cycle stiffness was not adopted because the swing phase contributes minimally to energy storage and utilization, and including it would dilute the functional relevance of stiffness to RE. For the average joint stiffness, the calculation formula was:

$$k_{\mathrm{stance}}=\frac{\Delta M}{\Delta \theta }$$ (4)

In Formula 4, $k_{\text{stance}}$ represents the average joint stiffness during the stance phase; ΔM represents the change in joint moment within the stance phase; and Δθ represents the change in joint angle within the same stance phase interval.

For RMS, the root mean square values of eight muscles were analyzed. For CAR, it included the muscle CAR of the hip, knee and ankle joints. It was calculated according to muscle RMS. The calculation formula was:

$${CAR}_{i}=\frac{{RMS}_{antagonist,i}}{{RMS}_{agonist,i}}$$ (5)

In Formula 5, CAR_i_ represents the muscle co-activation ratio of the *i*th joint, RMS_antagonist,i_ represents the EMG root mean square value of the antagonist muscle of the joint, and RMS_agonist,i_ represents the EMG root mean square value of the agonist muscle of the joint. RF and GM were the antagonist and agonist muscles of the hip joint, BF and VM were the antagonist and agonist muscles of the knee joint, and MG and TA were the antagonist and agonist muscles of the ankle joint (O’bryan et al., 2014).

### Statistical analysis

The Shapiro-Wilk test was used for normality test of the data. One-way repeated measures analysis of variance was used for RE, joint stiffness, muscle RMS and CAR under different PB speeds; paired samples t-test was used for joint PTR under different angular velocities; Pearson correlation analysis was used for correlation analysis of indicators. All parameters were expressed as mean ± standard deviation (Mean ± SD). The nominal level of statistical significance was set at p < 0.05.Given that a total of 100 Pearson correlation analyses were conducted, the family-wise error rate was controlled using the Bonferroni correction, yielding an adjusted significance threshold of p = 0.05/100 = 0.0005. Accordingly, for the Pearson correlation analyses, differences were considered statistically significant at p < 0.0005.However, after applying the Bonferroni correction (adjusted p < 0.0005), all correlation results were no longer statistically significant; therefore, the findings with p < 0.05 are interpreted as exploratory.

## Results

### Test indicator results

#### Aerobic capacity

The results of the aerobic capacity test are shown in Table 1. One-way repeated measures analysis of variance on the three RE values showed that there were significant differences among the three RE values (F = 63.576, p < 0.05, η^2^ = 0.876). Multiple comparison results showed that there were significant differences between each pair of RE values (p < 0.05).

**TABLE 1:** Results of aerobic capacity test.

VO_2_max (mL/kg/min)	RE70% (mL/kg/min)	RE80% (mL/kg/min)	RE90% (mL/kg/min)
53.36 ± 6.28	29.98 ± 2.58	36.21 ± 3.53^*^	43.11 ± 5.60^*#^

*indicates comparison with RE70%, p < 0.05; # indicates comparison with RE80%, p < 0.05.

#### Lower extremity explosive power

The results of the lower extremity explosive power test are shown in Table 2.

**TABLE 2:** Results of lower extremity explosive power test.

CMJ (cm)	SJ (cm)	EUR (%)	RSI (m/s)
33.09 ± 3.66	27.02 ± 2.44	5.72 ± 1.31	1.49 ± 0.20

GRF, data were processed with a 30 Hz low-pass fourth-order Butterworth filter.

#### Lower extremity joint PTR

The results of the lower extremity joint PTR test are shown in Table 3. Paired samples t-test on the test results of each joint at 60°/s and 180°/s found that in the flexor concentric-extensor concentric task, there was a significant difference in the knee joint between the two speeds (t = −4.902, p < 0.05); in the flexor concentric-extensor eccentric task, there was a significant difference in the knee joint between the two speeds (t = −2.369, p < 0.05).

**TABLE 3:** Results of lower extremity joint PTR test.

Joint Strength	PTR	Hip joint		Knee joint		Ankle joint	
		60°/s	180°/s	60°/s	180°/s	60°/s	180°/s
Static	Flexor concentric-extensor concentric	0.25 ± 0.09	0.30 ± 0.09	0.27 ± 0.03	0.31 ± 0.02	1.86 ± 0.41	1.99 ± 0.51
	Flexor eccentric-extensor eccentric	0.41 ± 0.08	0.40 ± 0.10	0.58 ± 0.07	0.55 ± 0.09	3.75 ± 0.96	3.47 ± 0.89
Dynamic	Flexor eccentric-extensor concentric	0.46 ± 0.08	0.41 ± 0.11	0.61 ± 0.08	0.62 ± 0.07	4.11 ± 0.71	4.67 ± 0.62
	Flexor concentric-extensor eccentric	0.28 ± 0.08	0.26 ± 0.10	0.30 ± 0.02	0.28 ± 0.03	1.37 ± 0.43	1.51 ± 0.52

#### Lower extremity stiffness and EMG test

**FIGURE 2:**
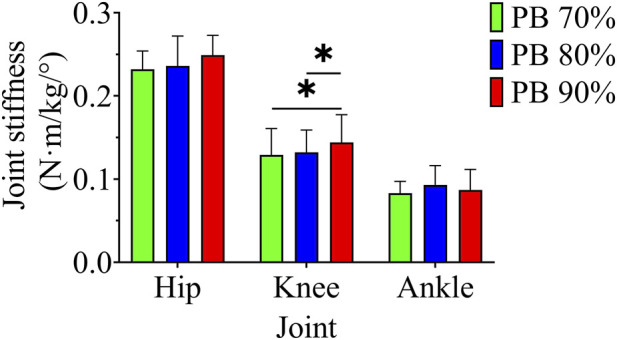
Joint stiffness under different speeds; * indicates p < 0.05.

The results of the lower extremity stiffness test are shown in Figure 2. One-way repeated measures analysis of variance on the stiffness of each joint at three speeds showed that there was a significant difference in the knee joint (F = 16.200, p < 0.05, η^2^ = 0.643). Multiple comparison results showed that the knee joint stiffness at PB70% and PB80% was significantly lower than that at PB90% (p < 0.05).

**FIGURE 3:**
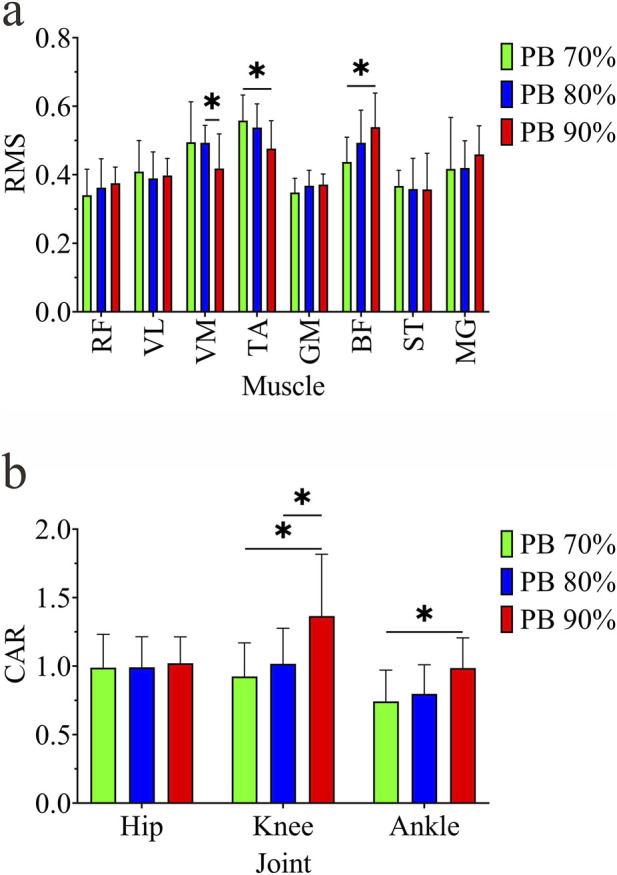
Muscle RMS and CAR under different speeds. **(a)** Muscle RMS; **(b)** CAR; * indicates p < 0.05.Note: EMG signals were normalized to the maximum value of each muscle across 3 MVC trials (each trial lasting 3–5 s, 60 s rest interval between trials).

The results of EMG RMS are shown in Figure 3a. One-way repeated measures analysis of variance on the RMS of each muscle at three speeds showed that there was a significant difference in VM (F = 5.456, p < 0.05, η^2^ = 0.286), and multiple comparison results showed that the RMS at PB80% was significantly higher than that at PB90% (p < 0.05); there was a significant difference in TA (F = 19.674, p < 0.05, η^2^ = 0.686), and multiple comparison results showed that the RMS at PB70% was significantly higher than that at PB90% (p < 0.05); there was a significant difference in BF (F = 6.328, p < 0.05, η^2^ = 0.413), and multiple comparison results showed that the RMS at PB70% was significantly lower than that at PB90% (p < 0.05).

The results of joint CAR are shown in Figure 3B. One-way repeated measures analysis of variance on the CAR of each joint at three speeds showed that there was a significant difference in the knee joint (F = 5.399, p < 0.05, η^2^ = 0.375), and multiple comparison results showed that the CAR at PB70% and PB80% was significantly lower than that at PB90% (p < 0.05); there was a significant difference in the ankle joint (F = 5.598, p < 0.05, η^2^ = 0.383), and multiple comparison results showed that the CAR at PB70% was significantly lower than that at PB90% (p < 0.05).

### Correlation analysis results

#### Correlation between aerobic capacity and PB

**FIGURE 4:**
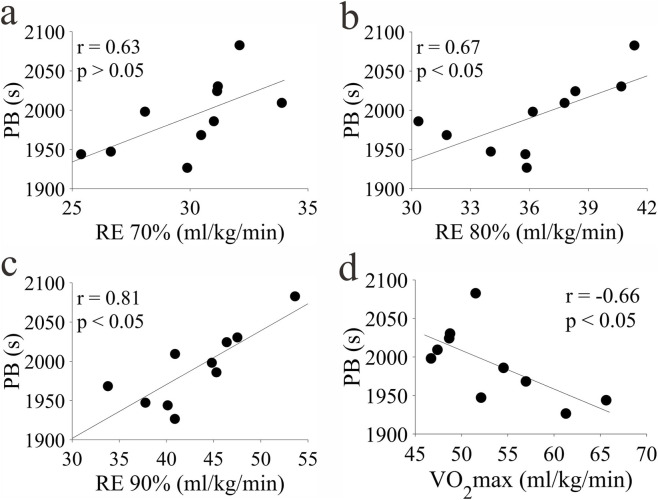
Correlation between aerobic capacity and PB. **(a-d)** represent RE70%, RE80%, RE90%, PB respectively.

The correlation results between aerobic capacity and PB are shown in Figure 4. With the increase of exercise intensity, the correlation between RE and PB gradually increased, and both RE80% and RE90% had a strong positive correlation with PB. At the same time, the correlation coefficients of the two groups exceeded that between VO_2_max and PB. This result indicates that RE is a key factor affecting long-distance running performance.

#### Correlation between lower extremity explosive power and RE, PB

**FIGURE 5:**
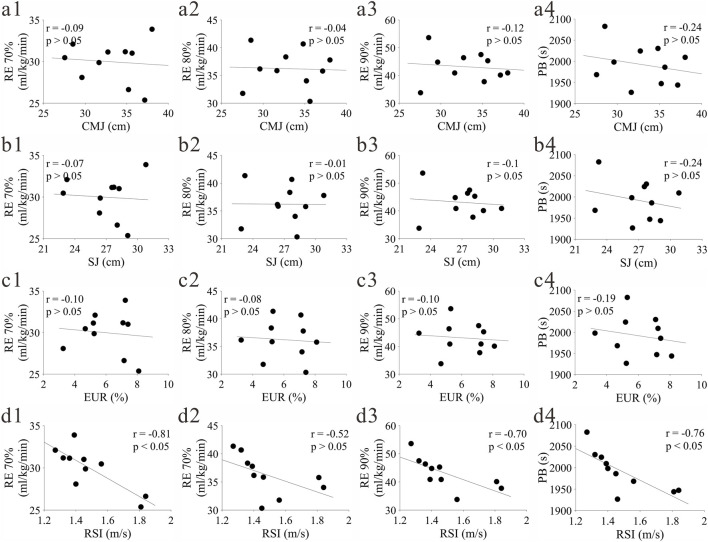
Correlation between lower extremity explosive power and RE PB. **(a–d)** represent CMJ, SJ, EUR, RSI respectively; (1-4) represent RE70%, RE80%, RE90%, PB respectively.

The correlation results between several lower extremity explosive power indicators and RE, PB are shown in Figure 5. No significant correlation was found between CMJ, SJ, EUR and RE, PB, but RSI showed a significant negative correlation with RE 70%, RE 90% and PB, indicating that a larger RSI is associated with better RE and PB, suggesting that RSI is an explosive power characteristic indicator that can better reflect RE and PB.

#### Correlation between joint PTR and RE, PB

**FIGURE 6:**
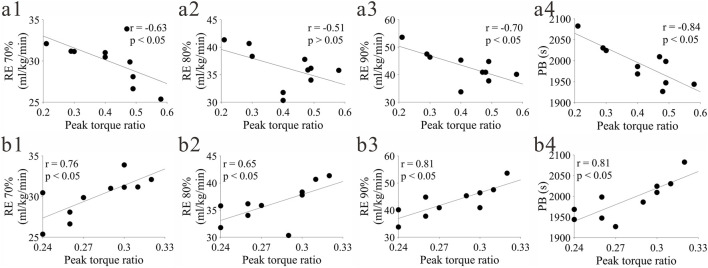
Significant correlation between joint PTR and RE. **(a)** represents hip joint flexor eccentric-extensor concentric at 180°/s speed; **(b)** represents knee joint flexor concentric-extensor eccentric at 180°/s speed; (1-4) represent RE70%, RE80%, RE90%, PB respectively.

At 180°/s speed, the hip joint flexor eccentric-extensor concentric was significantly positively correlated with three of the four indicators (RE 70%, RE90%, PB) (Figures 6a1–4), and the knee joint flexor concentric-extensor eccentric was significantly positively correlated with all four indicators (Figures 6b1–4). No significant correlation was found between other joint torque ratio results and RE, PB (p > 0.05).

#### Correlation between lower extremity joint stiffness and RE

**FIGURE 7:**
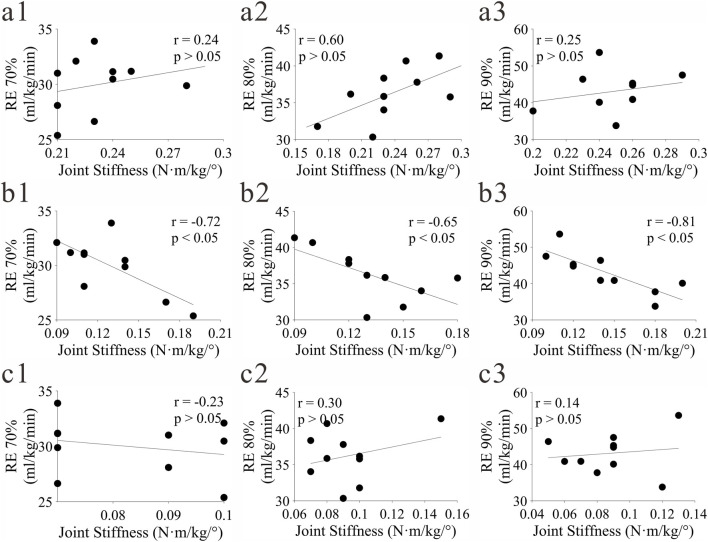
Correlation results between lower extremity stiffness and RE under different speeds. **(a–c)** represent hip, knee and ankle joints respectively; (1-3) represent RE70%, RE80%, RE90% respectively.

The correlation results between lower extremity stiffness and RE are shown in Figure 7. Among them, the knee joint stiffness at three speeds (PB70%, PB80%, PB90%) showed a significant negative correlation with the corresponding RE70%, RE80%, RE90%. No significant correlation was found between hip and ankle joint stiffness and RE (p > 0.05).

#### Correlation between RMS, CAR and RE

**FIGURE 8:**
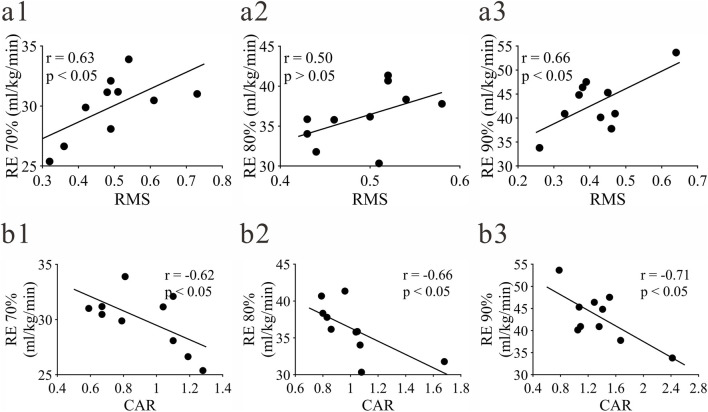
Significant correlation between RMS, CAR and RE. **(a)** represents the RMS of VM; **(b)** represents the CAR of the knee joint; (1-3) represent RE70%, RE80%, RE90% respectively.

In terms of RMS indicators, VM showed a positive correlation with RE (Figures 8a1–3), and a significant correlation with the corresponding RE70% and RE90% at PB70% and PB90% speeds. No significant correlation was found between RMS of other muscles and RE (p > 0.05).

In terms of CAR indicators, the knee joint CAR at three speeds (PB70%, PB80%, PB90%) showed a significant negative correlation with the corresponding RE70%, RE80%, RE90% (Figures 8b1–3). No significant correlation was found between hip and ankle joint CAR and RE (p > 0.05).

## Discussion

This exploratory, hypothesis-generating study took male second-class long-distance runners as the research objects, and systematically explored the potential correlation mechanisms between lower extremity explosive power, strength balance, joint stiffness, neuromuscular characteristics and RE as well as personal best (PB) under different relative paces. A total of 100 Pearson correlation tests were conducted, and the Bonferroni correction was applied to control the risk of false-positive findings (adjusted p < 0.0005). None of the correlations that were significant before correction remained statistically significant after correction, which should be attributed to the large number of tests and the relatively small sample size. Therefore, all correlation-related findings in this study are strictly exploratory and hypothesis-generating, and no definitive causal or correlational conclusions can be drawn. The results showed that RE increased significantly with the increase of pace, and the correlation between RE and PB at medium and high intensity paces (PB80%, PB90%) was better than that of maximal oxygen uptake (VO_2_max), which provides preliminary clues for confirming RE as a core indicator associated with long-distance running performance. At the same time, RSI, joint PTR at specific angular velocities, knee joint stiffness and neuromuscular activation characteristics (VM, RMS, knee joint CAR) showed potential associations with RE and PB before correction, providing key preliminary empirical support for generating hypotheses about the regulatory mechanism of running energy efficiency.

### Correlation between RE and pace, sports performance

This study found that RE showed a significant gradient increase at three paces (PB70%, PB80%, PB90%), which is consistent with the basic law of increasing energy consumption with intensity in long-distance running. As the pace increased from PB70% to PB90%, the body needed to mobilize more muscle fibers to participate in contraction, and the oxidation rate of metabolic substrates accelerated, ultimately resulting in a significant increase in oxygen uptake. It is worth noting that the correlation coefficients between RE80%, RE90% and PB were higher than those between VO_2_max and PB. This result tentatively supports the classic view that “among athletes with similar VO_2_max, RE is a key factor distinguishing competitive levels”, but due to the small sample size, this conclusion needs to be validated by larger-scale studies (Daniels and DANIELS, 1992). For the second-class athlete group, VO_2_max has reached a certain level, while RE may have greater room for optimization. Therefore, improving RE through targeted training may potentially become an important breakthrough for improving long-distance running performance, but this needs to be confirmed by subsequent intervention studies.

### Influence of lower extremity explosive power characteristics on RE

The correlation results between lower extremity explosive power and RE showed that RSI was significantly negatively correlated with RE70%, RE90% and PB, while CMJ, SJ and EUR had no significant correlation with the above indicators. This finding is consistent with some research conclusions, indicating that RSI can better reflect the energy storage and feedback efficiency of the muscle-tendon system in long-distance running (Tanneau et al., 2025). RSI comprehensively reflects the conversion speed and energy utilization efficiency of muscle eccentric contraction to concentric contraction in the SSC. A higher RSI is associated with better RE and PB, which may be due to athletes being able to release elastic potential energy more quickly during running, thereby reducing metabolic energy consumption.

The lack of significant correlation between CMJ, SJ and EUR may be due to two reasons: first, the participants in this study were all second-class athletes with similar training levels, and the individual differences in basic explosive power indicators such as CMJ and SJ were small, making it difficult to reflect the correlation with RE; second, as the difference ratio between CMJ and SJ, EUR more reflects the elastic energy utilization in static vertical jump, while the elastic potential energy conversion in long-distance running depends on dynamic step frequency and stride adjustment and neural control timing, which is different from the mechanical characteristics of static testing (Komi, 2000). This suggests that RSI may be more specific and sensitive than traditional vertical jump height indicators in evaluating the explosive power-related energy efficiency of long-distance runners, but this finding is preliminary due to the limited statistical power of the study, and further verification with expanded sample sizes is required.

### Correlation between lower extremity strength balance and RE

The analysis results of joint PTR showed that at 180°/s angular velocity (representing speed strength capacity), the hip joint flexor eccentric-extensor concentric PTR was significantly positively correlated with RE70%, RE90% and PB, and the knee joint flexor concentric-extensor eccentric PTR was significantly positively correlated with RE at all paces and PB, while no similar correlation was found at 60°/s angular velocity (representing maximal strength capacity). This result indicates that the association between strength balance and running energy efficiency is significantly speed-dependent, and the speed strength balance in dynamic movement is more closely related to RE.

The 180°/s angular velocity is close to the joint movement speed during long-distance running. The PTR under this condition reflects the cooperative work efficiency of flexor and extensor muscles of athletes during dynamic running. The optimization of hip joint flexor eccentric-extensor concentric PTR may help to improve the coordination efficiency of lower limb forward swing and push-off during the swing phase; the rationality of knee joint flexor concentric-extensor eccentric PTR may reduce joint stress concentration during support and lower energy waste (Wight et al., 2022). However, the PTR at 60°/s angular velocity focuses more on the balance of static maximal strength, which has a low matching degree with the dynamic movement characteristics of long-distance running, so no significant correlation was shown. This tentatively suggests that in long-distance running strength training, emphasis may be placed on the strength balance of flexor and extensor muscles at medium and high angular velocities, rather than simply pursuing the symmetry of maximal strength. However, this conclusion is based on a small sample size and needs to be validated by studies with larger samples and more diverse populations.

### Regulatory role of joint stiffness on RE

The analysis results of joint stiffness showed that the knee joint stiffness at three paces was significantly negatively correlated with the corresponding RE, while the hip and ankle joint stiffness had no significant correlation with RE. This finding preliminarily indicates that knee joint stiffness may be a key mechanical parameter associated with running energy efficiency, and moderately increasing knee joint stiffness may help to improve RE. Due to the insufficient statistical power of the study, this regulatory relationship needs to be further confirmed by studies with larger sample sizes and more comprehensive test designs. As the core hub of lower extremity movement, the knee joint bears the main ground reaction force during the support phase. Moderate stiffness can optimize the efficiency of elastic potential energy storage and release and reduce muscle metabolic burden; excessively low stiffness will lead to energy leakage, and excessively high stiffness will increase impact absorption cost, both of which will reduce RE (Tam et al., 2019).

The lack of a significant correlation between hip/ankle joint stiffness and RE may be mediated by three mechanisms. First, the homogeneous training background of the participants induced adaptive homogenization of Achilles tendon mechanical properties (e.g., compliance and Young’s modulus) (Chen et al., 2020), minimizing inter-individual variability in ankle joint stiffness—an essential prerequisite for correlation analysis. Second, the hip joint’s functional positioning (focused on lower limb swing and trunk stability) leads to stable stiffness across paces (Dumke et al., 2010), with its regulation prioritizing gait coordination over energy efficiency (Goldberg and Neptune, 2007). Third, the coupling between ankle stiffness and RE depends on tendon-muscle synergy (Ettema, 1996); similar Achilles tendon properties among participants restrict the amplitude of stiffness adjustment, preventing significant differences in RE. Studies on heterogeneous runner groups have confirmed that distinct tendon properties strengthen the stiffness-RE correlation (Liu et al., 2022), supporting the above mechanisms.

### Influence of neuromuscular activation characteristics on RE

Among the neuromuscular activation indicators, the root mean square (RMS) of vastus medialis (VM) was significantly positively correlated with RE70% and RE90%, and the knee joint co-activation ratio (CAR) was significantly negatively correlated with RE at all paces, revealing the important regulatory role of neuromuscular coordination strategies on RE. As an important part of the knee joint extensor muscles, the reasonable increase of VM activation intensity (RMS) may enhance knee joint support stability and push-off efficiency, thereby reducing energy consumption per unit distance. This is a preliminary observation, and the reliability of this mechanism needs to be verified by more in-depth studies with larger samples (Guzmán-Venegas et al., 2021). However, the RMS of VM at PB80% was significantly higher than that at PB90%, which may be because PB80% is in the athletes’ “efficient pace range”, where muscle activation and energy consumption reach the best balance, while PB90% is close to the exhaustion intensity, and muscle fatigue leads to decreased activation efficiency.

The significant negative correlation between knee joint CAR and RE is consistent with the theoretical hypothesis that “low co-activation ratio can reduce ineffective consumption of antagonist muscles”. A lower CAR may mean that the nervous system can effectively inhibit the redundant activities of antagonist muscles, making the contraction energy of agonist muscles more concentrated on propulsive movement, thereby improving RE. This conclusion is based on correlation analysis with a small sample size, and causal verification through intervention studies is required (Dal Maso et al., 2012). As the pace increased to PB90%, the knee joint CAR increased significantly, which may be a regulatory strategy adopted by the body to maintain joint stability during high-speed running, but excessively high CAR will also lead to increased metabolic cost, which is consistent with the result that RE reached its peak at PB90%. The lack of significant correlation between hip and ankle joint CAR may be related to the functional positioning of these joints in long-distance running, and their co-activation regulation focuses more on stability guarantee rather than energy efficiency optimization.

In addition, the present study observed a decrease in VM and TA muscle activity (RMS) at the highest intensity (PB90%) compared to lower paces, which seems to contradict the “size principle” of motor unit recruitment and the general understanding that muscle activation increases with exercise intensity. This counterintuitive finding may be explained by two key mechanisms:

First, PB90% is close to the participants’ maximal exercise intensity (approaching VO_2_max), and prolonged high-intensity exercise leads to neuromuscular fatigue (FIORENZA et al., 2019). Previous studies have shown that when exercise intensity exceeds the individual’s lactate threshold, the accumulation of metabolic byproducts (e.g., lactic acid) and the depletion of energy substrates (e.g., ATP) can impair the excitability of motor neurons and the coupling efficiency between nerve and muscle (Sale, 1988), resulting in a decrease in muscle activation intensity despite the body’s attempt to maintain high motor unit recruitment. For VM (a key knee extensor) and TA (responsible for ankle dorsiflexion and gait coordination), their continuous participation in supporting and propulsive movements during high-speed running leads to more rapid fatigue accumulation, which may explain the significant decrease in RMS at PB90%.

Second, the “size principle” primarily describes the sequential recruitment of motor units under submaximal, non-fatigued conditions (Henneman et al., 1965). However, during near-maximal intensity running, the nervous system may adopt a functional optimization strategy instead of simply increasing activation intensity. At PB90%, the body prioritizes maintaining gait stability and propulsive efficiency by adjusting the coordination pattern of agonist-antagonist muscles rather than enhancing the activation of individual muscles. For TA, its main function is to coordinate ankle movement during the swing phase; at ultra-high speeds, the shortening of the swing phase may reduce the demand for TA activation, while the nervous system shifts its regulatory focus to core stability and knee joint propulsion (Kyröläinen et al., 2001). This functional redistribution of neuromuscular control may lead to a decrease in the activation of specific muscles (VM, TA) despite overall high exercise intensity, which may not contradict the essence of the size principle but reflect its adaptive adjustment under fatigue and high-intensity conditions. This mechanism is a preliminary hypothesis derived from a small sample study, and further verification with larger samples and more detailed physiological measurements is needed.

A notable apparent inconsistency exists between the between-speed trend of knee joint CAR and its within-speed correlation with RE: across paces, CAR increases significantly with increasing intensity, while within each speed, CAR shows a significant negative correlation with RE. This inconsistency can be explained by two interconnected mechanisms rooted in the pace-dependent regulation of neuromuscular coordination:

First, the between-speed increase in CAR reflects a functional adaptation to maintain joint stability under high-intensity running. As pace rises to PB90%, the ground reaction force and joint movement velocity increase substantially, requiring the nervous system to enhance co-activation of agonist and antagonist muscles around the knee joint to prevent excessive joint displacement and reduce injury risk (Jiang et al., 2016). This stability-prioritized regulation leads to a mandatory increase in CAR, which is a necessary trade-off for maintaining gait integrity at high speeds.

Second, the within-speed negative correlation between CAR and RE reveals individual differences in the efficiency of this stability-optimization strategy. For runners with the same pace (and thus similar stability demands), a lower CAR indicates more efficient neural control—i.e., the nervous system inhibits redundant antagonist muscle activation while ensuring joint stability (Dal Maso et al., 2012). This reduces metabolic waste from ineffective muscle contraction, thereby improving RE. In contrast, runners with higher CAR at the same pace exhibit excessive co-activation that does not contribute to additional stability but increases energy consumption, resulting in poorer RE.

In summary, the between-speed CAR increase is a universal adaptive response to escalating stability requirements, while the within-speed negative correlation reflects individual variability in neuromuscular coordination efficiency. This apparent inconsistency underscores the dual role of CAR in running: it is both a necessary stability mechanism (explaining the upward trend with pace) and a key determinant of energy efficiency (explaining the negative correlation with RE at each pace).

### Considerations on conceptual overlap among variables

The measured variables in this study, including RSI, joint stiffness, EMG RMS, CAR, and PTR, essentially revolve around the core physiological mechanisms of neuromuscular control strategies and elastic energy utilization, leading to inevitable conceptual overlap. For instance, RSI reflects the rapid energy storage and release efficiency of the tendon-muscle system, while knee joint stiffness directly affects the storage and conduction of elastic potential energy, both of which are closely related to stretch-shortening cycle (SSC) function. EMG RMS and CAR collectively reflect the neural regulation of muscle activation: the former indicates muscle activation intensity, and the latter reflects the coordination between antagonist and agonist muscles, ultimately influencing running economy (RE) by affecting muscle contraction efficiency. Similarly, joint PTR at 180°/s angular velocity not only reflects the strength balance of muscle groups but also indirectly reflects the neural regulation of muscle synergy during dynamic movements, sharing functional relevance with neuromuscular activation indicators.

It should be explicitly clarified that the correlation analysis of individual variables in this study only aims to preliminarily reveal potential associations between single indicators and RE or sports performance, rather than interpreting them as independent regulatory mechanisms. The associative effects of each variable essentially reflect the synergistic action of the neuromuscular-mechanical system, and no single indicator can fully and independently explain the variation in RE. Based on this, the conclusions of this study emphasize “potential associations” and “exploratory hypotheses,” avoiding overinterpretation of the independent role of individual variables, and merely providing preliminary reference clues for subsequent targeted training.

## Research limitations and future directions

This study has several notable limitations that need to be acknowledged. First, the sample size is small (n = 10) while a large number of outcome variables were examined, including RE at three intensities, four explosive power indices (CMJ, SJ, EUR, RSI), multiple PTR measures across different joints and angular velocities, joint stiffness of three lower extremity joints, EMG RMS of eight muscles, CAR indices of three joints, and PB. This results in insufficient statistical power, which increases the risk of both Type I (false positive) and Type II (false negative) errors. Significant correlations identified in this study may be unstable and sensitive to outliers, while non-significant findings may reflect insufficient power rather than the true absence of effects. Second, the study sample is restricted to male second-class long-distance runners from a single institution, which greatly limits the generalizability of the results. The findings may not be applicable to female runners, athletes of different performance levels (e.g., elite or recreational runners), or those from different training backgrounds and regions. Third, the test only covers three relative paces (PB70%, PB80%, PB90%) and does not involve changes in indicators after ultra-high intensity or long-duration endurance exercise, making it difficult to reflect the regulatory mechanism of RE under fatigue conditions. Fourth, the impact of training intervention on related indicators is not explored, and the causal relationship cannot be clarified based on the cross-sectional correlation analysis. Fifth, theoretically, inter-limb asymmetry may lead to gait imbalance, increase energy loss, and reduce RE (Blagrove et al., 2021). However, considering that all participants in this study are second-class athletes with long-term systematic training, their motor control ability and limb symmetry are already at a high level. Therefore, this study did not evaluate the potential impact of inter-limb asymmetry.

Future research can be carried out from three aspects: first, expand the sample size to include runners of different genders, ages and sports levels from multiple institutions to improve statistical power and verify the generalizability of the conclusions of this study; second, add test scenarios under fatigue conditions to explore the association between neuromuscular and mechanical characteristics and RE after long-term exercise; third, carry out intervention studies, design targeted training programs based on the key indicators found in this study (such as RSI, knee joint stiffness, specific PTR, etc.), and verify their effects on improving RE and long-distance running performance.

## Conclusion

This study is exploratory and hypothesis-generating. A total of 100 Pearson correlation tests were conducted, and the Bonferroni correction was applied (adjusted p < 0.0005), with none of the previously significant correlations retaining statistical significance after correction. Nevertheless, preliminary exploratory findings showed that RE increases with the increase of pace, and the correlation with PB at medium and high speed paces is stronger than that of maximal oxygen uptake; among lower extremity explosive power indicators, only RSI showed a potential negative association with RE and PB before correction, while CMJ, SJ and EUR had no obvious correlational patterns; at 180°/s angular velocity, the hip joint flexor eccentric-extensor concentric and knee joint flexor concentric-extensor eccentric PTRs showed potential correlations with RE and PB before correction, but there was no such trend at 60°/s angular velocity; knee joint stiffness showed a potential negative association with RE at all paces before correction, suggesting it may be a key mechanical parameter potentially related to RE; the RMS of VM showed a potential positive association with RE, and the knee joint CAR showed a potential negative association with RE at all paces before correction, indicating both may be jointly involved in the regulatory patterns of RE. The potential associations between these indicators and RE are pace-dependent and joint-specific, which can provide preliminary exploratory clues and theoretical references for generating hypotheses about targeted long-distance running training and improving competitive performance. It should be emphasized that due to the lack of statistical significance after Bonferroni correction, the conclusions of this study are strictly preliminary and exploratory. No causal inferences can be drawn due to the cross-sectional design and small sample size, and future studies with larger sample sizes and rigorous designs are needed to verify these potential associations.

## Data Availability

The original contributions presented in the study are included in the article/Supplementary Material, further inquiries can be directed to the corresponding author.
